# Gene Network and Pathway Analysis of Mice with Conditional Ablation of Dicer in Post-Mitotic Neurons

**DOI:** 10.1371/journal.pone.0044060

**Published:** 2012-08-27

**Authors:** Véronique Dorval, Pascal Y. Smith, Charlotte Delay, Ezequiel Calvo, Emmanuel Planel, Nadège Zommer, Luc Buée, Sébastien S. Hébert

**Affiliations:** 1 Axe Neurosciences, Centre de Recherche du CHUQ (CHUL), Québec, Québec, Canada; 2 Département de Psychiatrie et de Neurosciences, Université Laval, Québec, Québec, Canada; 3 Université Lille-Nord de France, UDSL, Faculté de Médecine, Lille, France; 4 Inserm, UMR837, Lille, France; Cardiovascular Research Institute Maastricht, The Netherlands

## Abstract

**Background:**

The small non-protein-coding microRNAs (miRNAs) have emerged as critical regulators of neuronal differentiation, identity and survival. To date, however, little is known about the genes and molecular networks regulated by neuronal miRNAs *in vivo*, particularly in the adult mammalian brain.

**Methodology/Principal Findings:**

We analyzed whole genome microarrays from mice lacking *Dicer*, the enzyme responsible for miRNA production, specifically in postnatal forebrain neurons. A total of 755 mRNA transcripts were significantly (P<0.05, FDR<0.25) misregulated in the conditional *Dicer* knockout mice. Ten genes, including Tnrc6c, Dnmt3a, and Limk1, were validated by real time quantitative RT-PCR. Upregulated transcripts were enriched in nonneuronal genes, which is consistent with previous studies *in vitro*. Microarray data mining showed that upregulated genes were enriched in biological processes related to gene expression regulation, while downregulated genes were associated with neuronal functions. Molecular pathways associated with neurological disorders, cellular organization and cellular maintenance were altered in the *Dicer* mutant mice. Numerous miRNA target sites were enriched in the 3′untranslated region (3′UTR) of upregulated genes, the most significant corresponding to the miR-124 seed sequence. Interestingly, our results suggest that, in addition to miR-124, a large fraction of the neuronal miRNome participates, by order of abundance, in coordinated gene expression regulation and neuronal maintenance.

**Conclusions/Significance:**

Taken together, these results provide new clues into the role of specific miRNA pathways in the regulation of brain identity and maintenance in adult mice.

## Introduction

Accumulating evidence suggest that miRNAs, highly conserved small noncoding regulatory RNAs, are required for cell differentiation, identity, and maintenance [1], [2]. Like protein-coding genes, miRNA genes are embedded in the genome and are mostly transcribed by the RNA polymerase II [3]. In the cytoplasm, the precursor miRNA is cleaved by the ribonuclease Dicer to generate ∼21 nucleotide double-stranded RNAs. The mature miRNA is then loaded into the RNA-induced silencing complex (RISC), which comprises Dicer and the Argonaute (eif2c/Ago) proteins as the catalytic core [4]. As part of this complex, single-stranded miRNAs target, with partial complementarity, mRNA transcripts mainly within the 3′UTR leading to mRNA degradation or translational repression [5]. Nucleotides 2–8 from the 5′ end of the mature miRNA, known as the seed region, are important for this targeting [6]. Each miRNA can target up to several hundred mRNAs *in vivo*, therefore potentially regulating multiple biological pathways [7].

In the brain, several miRNAs are specifically expressed during development [8]–[12]. Studies inactivating *Dicer* demonstrate that miRNAs in general are essential for mammalian brain morphogenesis [13] as well as post-mitotic neuronal survival [14], [15]. One of the most conserved and abundant brain miRNAs, miR-124, can stimulate neuronal differentiation both *in vitro* and *in vivo* by targeting the transcriptional repressor REST, a negative regulator of neurogenesis [16]–[19]. Introduction of miR-124 in nonneuronal HeLa cells converts the overall gene-expression pattern to a neuronal one [20]. Conversely, inhibition of endogenous miR-124 in cultured primary neurons results in an accumulation of nonneuronal transcripts [17]. Thus, in cells undergoing neuronal differentiation, specific miRNAs can fine-tune the transcriptome towards that of a terminally differentiated cell type. Whether miR-124 (and possibly REST) functions in neuronal maintenance *in vivo* remains unexplored.

Apart from miR-124, several miRNAs play significant roles in the neuron [21]. For instance, miR-132 and miR-134 have been implicated in neuronal outgrowth and synaptic plasticity, respectively [22], [23]. It remains unclear however to what extent these or other miRNAs participate in neuronal maintenance *in vivo*. In addition, the gene networks and pathways dependent on miRNA activity in the adult mammalian brain remain largely unknown. In order to address these issues, we studied genome-wide transcriptional profiles associated with miRNA depletion in mouse postnatal forebrain neurons. We identified several important biological pathways that were affected in the Dicer-deficient mice. These were associated with, for instance, gene expression regulation, neuronal function, and cell integrity. Notably, more than 60% of upregulated genes were enriched in specific miRNA seed sequences, including miR-124 as well as many other miRNAs, highlighting the potential physiological importance of these miRNAs in the neuron. Overall, this study confirms and extends previous observations suggesting that miR-124 plays an important role in neuronal identity and maintenance *in vivo*. Moreover, and importantly, our *in silico* analyses suggest that miR-124 functions in concert with a large fraction the miRNome to regulate neuronal homeostasis.

## Results

### Global transcriptome analysis of mice lacking Dicer in post-mitotic neurons

We performed genome-wide microarrays (Affymetrix mouse exon ST 1.0) on brain tissue isolated from 9–10.5 week-old *Dicer* conditional knockout (cKO) mice [14]. In these mice, neuronal Dicer inactivation (i.e., removal of the second RNase domain) was achieved using a forebrain-specific α-CamKII promoter [13]. This model displays decreased levels of mature miRNAs in the brain, including the neuron-specific miR-124, and shows no signs of apoptosis in the cortex, our region of interest. At the age of study, the *Dicer* mutant mice start showing signs of hypoactivity and impaired social interaction, as previously documented [14].

**Figure 1:**
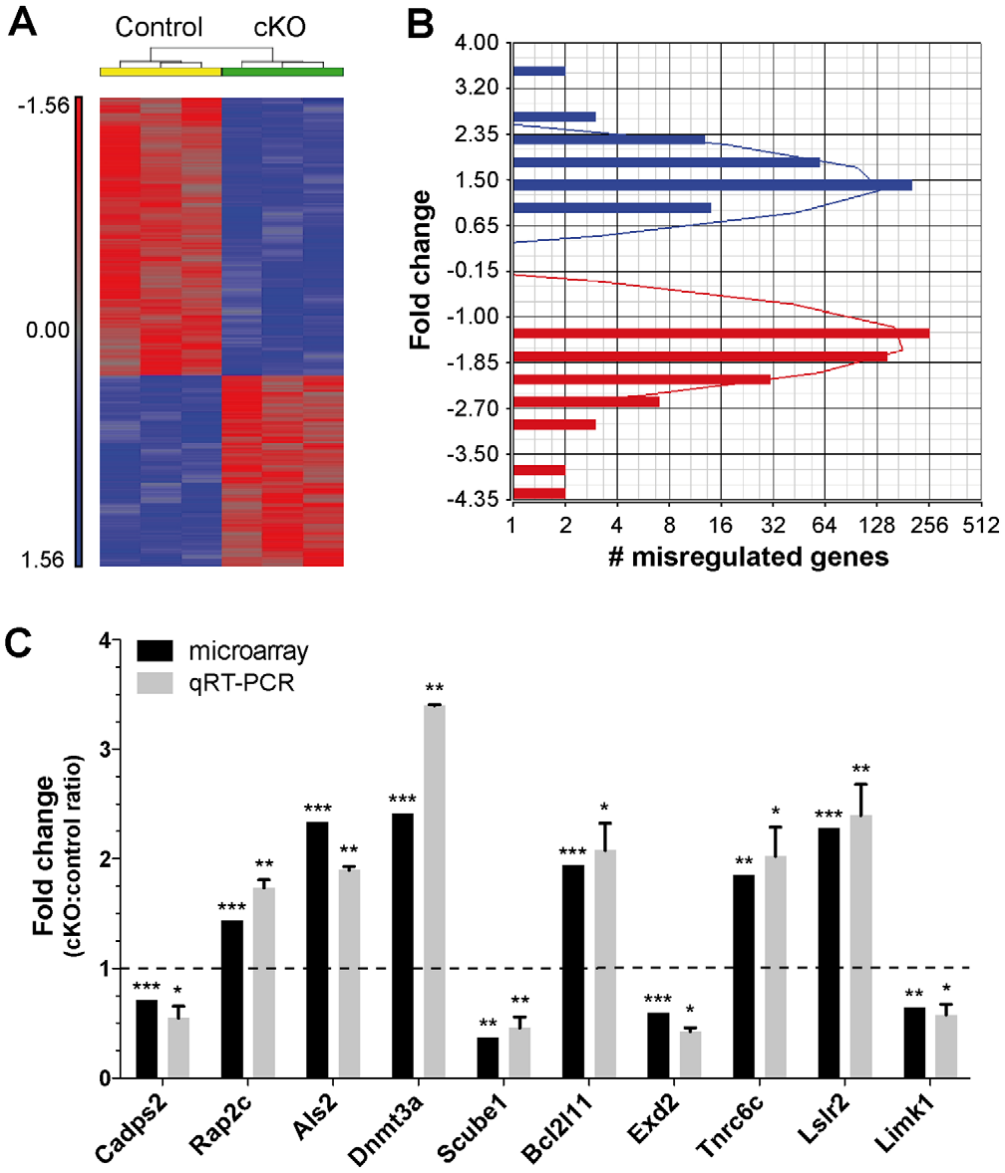
Gene expression changes in the absence of neuronal Dicer *in vivo*. (**A**) Cluster analysis of microarray data from control and *Dicer* cKO mice (cortex). Here, all genes significantly changed (P<0.05, FDR <0.25, n = 798) were included in this analysis. Results were generated using Partek Genomics Suite. (**B**) Histogram showing that 65–75% of misregulated transcripts has less than 1.5-fold difference in gene expression in the *Dicer* mutant mice when compared to controls. *y*-Axis  =  log2(x). (**C**) Validation of selected genes by real-time quantitative RT-PCR. Gapdh was used as normalization control. Statistical significance was determined by a *Student unpaired t* test (* = p<0.05, ** = p<0.01, *** = p<0.001). Standard deviation is shown.

Using microarrays, we identified 755 transcripts to be significantly (P<0.05, FDR <0.25) altered in the *Dicer* cKO mice when compared to controls (Figure 1A and Table S1). From those transcripts, 40% (299) and 60% (456) were upregulated and downregulated, respectively. Interestingly, the majority (65–70%) of transcripts had less than 1.5-fold difference in expression levels (Figure 1B), which is consistent with the notion that miRNAs are mainly involved in the fine-tuning of gene expression [24]. It should be cautioned, however, that cre-negative cells (e.g., glia) might also reduce the overall probe signals on the microarray chips.

By real-time quantitative RT-PCR (qRT-PCR), we confirmed changes in 10 selected candidate genes (Figure 1C). These genes were chosen based on their high p-values and relevance to our biological pathways of interest (see below). These include the upregulated genes *Rap2c*, *Tnrc6c*, *Bcl2l11*, *Als2*, *lslr2*, and *Dnmt3a*, as well as the downregulated genes *Cadps2*, *Limk1*, *Exd2*, and *Scube1*. Primers for each gene are listed in the Table S2.

### Biological functions and tissue profiles associated with neuronal miRNA loss in vivo

We next performed microarray data mining using the DAVID v6.7 logarithm [25]. This analysis showed that misregulated genes (n = 755) were significantly enriched for various biological processes (Table S3). When analyzed separately, upregulated genes (n = 299) were significantly enriched for biological processes involved in, for instance, gene expression regulation, including among the 6 top-ranked gene ontology (GO) terms *transcription* (P = 4.77e–06), *chromatin organization* (P = 1.02e–05), *chromatin modification* (P = 2.23e–5), and *negative regulation of transcription from the RNA polymerase II promoter* (P = 1.09e–3) (Table S3). Interestingly, key genes associated with the miRNA machinery were included in the set of upregulated genes, including *Dicer1* itself, *Ago2/Eif2c2*, and *Tnrc6c* (Table S1). The latter gene encodes for a protein that interacts with Ago isoforms and is involved in miRNA-mediated mRNA repression [26]. These changes could possibly represent the loss of a negative feedback loop between Dicer and miRNA activity [27]. In contrast, downregulated genes (n = 456) were enriched for biological processes highly, but not exclusively, associated with neuronal function, including the GO terms *regulation of axonogenesis* (P = 1.94e–04), *regulation of neuron projection development* (P = 5.52e–04), and *regulation of axon extension* (P = 4.82e–03) (Table S3).

We also noticed that upregulated transcripts were associated with nonneuronal tissue profiles, primarily *embryonic tail* (P = 5.82e–07) and *macrophages* (P = 1.31e–04), whereas downregulated genes were associated with the tissue of study, that is, *brain* (P = 1.61e–11) followed by *cortex* (P = 3.82e–08) (Table S3). Thus, and consistent with previous studies [17], [20], Dicer deficiency *in vivo* is associated with signs of neuronal identity and function loss.

### Analysis of molecular pathways in the Dicer cKO brain

We next used the Ingenuity Pathway Analysis (IPA) tool (see materials and methods) to search for molecular pathways associated with neuronal miRNA loss in mammals. Here, all misregulated genes (i.e., upregulated and downregulated) were included in the analysis. Top biological functions, canonical pathways and gene networks that are affected in the neuronal *Dicer* mutant mice are provided in Table S4. High-ranking pathways include the terms *neurological disease* (P = 9.74e–11), *cellular assembly and organization* (P = 9.96e–08) as well as *nervous system development and function* (P = 3.09e–05).

**Figure 2:**
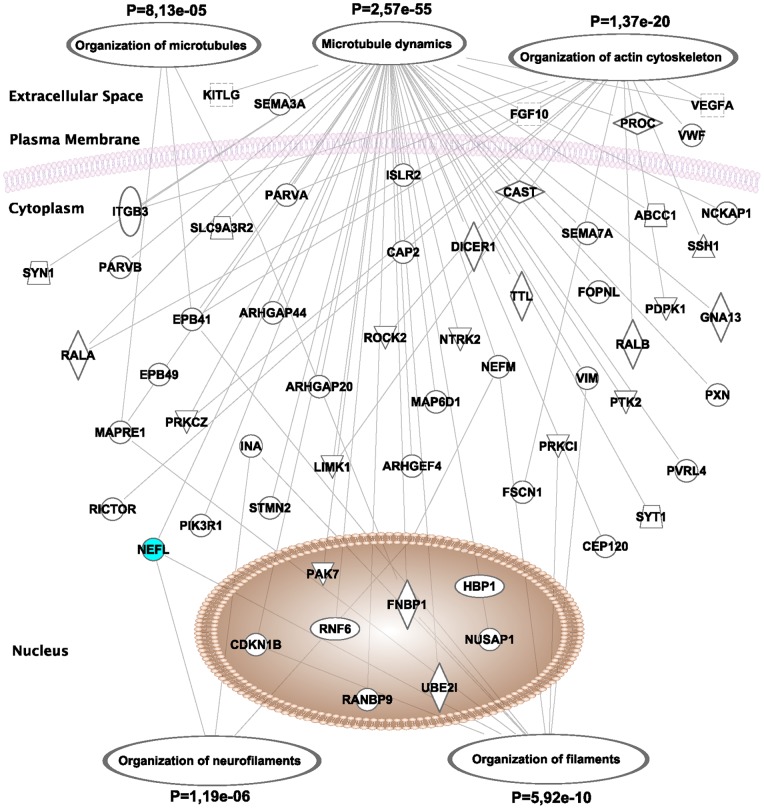
Significant gene networks associated with neuronal miRNA loss in post-mitotic neurons. (**A**) Shown here are IPA-generated pathways. Both upregulated and downregulated genes were included in the analysis. Significant biological functions are associated with the regulation of the cytoskeleton. Relationships are primarily due to co-expression, but can also include phosphorylation/dephosphorylation, proteolysis, activation/deactivation, transcription, binding, inhibition, and biochemical modification. Please refer to Figure S1 for further details. Nefl downregulation (in blue) was confirmed at the protein level in Figure 3. P values were calculated by IPA.

**Figure 3:**
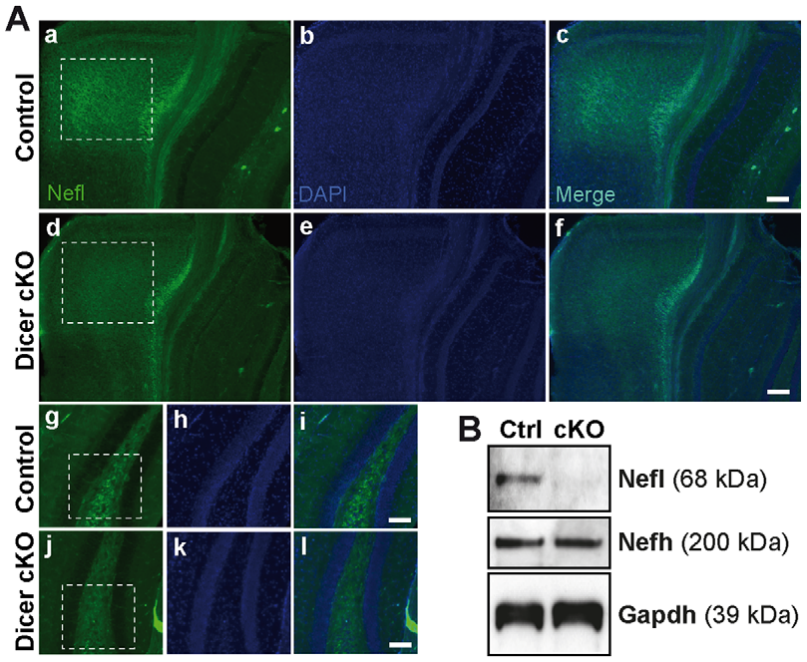
Validation of neurofilament changes in the *Dicer* cKO mice. (**A**) Immunohistochemistry of Nefl in the cortex (a–f) and the dentate gyrus (g–l) of control (a–c, g–i) and *Dicer* cKO (d–f, j–l) mice. Note the reduction in Nefl signal (in green) in the mutant mice (highlighted in white square). Of mention, changes in the cortex were more pronounced in 13 week-old *Dicer* cKO mice (shown here) when compared to age-matched controls. Dentate gyrus stainings gave similar results in both 9–10.5 and 13 week-old mice (9.5 week-old mouse shown here). DAPI (nuclei) stainings are shown in blue. Scale bars 20 µm (a–l). (**B**) Representative (n = 5) western blot analysis of Nefl and Nefh in cortex samples of 9–10.5 week-old control and *Dicer* cKO mice. Note that only Nefl was downregulated in the *Dicer* cKO mice. Gapdh was used as internal loading control.

One salient feature associated with Dicer loss in the cortex is neuronal shrinkage [14], [28]. Interestingly, the LIM domain kinase 1 (Limk1), one of the validated genes (downregulated), encodes an abundant neuronal protein that regulates various aspects of the cytoskeleton related to the organization of actin filaments and is important for the maintenance of cellular size and shape [29]. Moreover, several sub-networks associated with terms such as *microtubule assembly and neurofilament organization* were affected in the *Dicer* mutant mice (Figure 2 and Figure S1). Other candidate effector genes included *tubulin* (−1.67 fold, P = 2.75e–3), the building block of microtubules, as well as *neurofilament light and medium polypeptides (Nefl and Nefm)* (−1.68 fold, P =  6.680e–3 and −1.67 fold, P = 2.85e–3), all of which were reduced in the mutant mice. By immunohistochemistry (Figure 3A) and western blot (Figure 3B), we could show that Nefl protein levels were reduced in the *Dicer* cKO mice when compared to controls, which is consistent with the microarrays. In contrast, and in accordance with our profiling data, we detected no significant changes in *neurofilament heavy polypeptide* (Nefh) protein levels in the *Dicer* mutant mice (Figure 3B and Figure S2). Combined with our previous studies involving the microtubule-associated protein tau [14], these genes could potentially contribute to the neuronal shrinkage phenotype in the *Dicer* cKO mice.

DNA methylation is an epigenetic mechanism important for modulating gene expression, which is in accordance with the DAVID results (Table S3). A change in the DNA methyltransferase *Dnmt3a* (2.41 fold, P = 5.56e–4) is thus of particular interest (Table S1 and Figure 1C). Among miRNAs involved in *Dnmt3a* regulation, miR-29 is a prime candidate [30]. While expressed in post-mitotic neurons, the role of this enzyme in the brain, however, remains controversial. One study reported that DNA methylation is important for synaptic function [31], while another study showed that epigenetic mechanisms drive neuronal apoptosis, at least in motor neurons [32]. Further studies are therefore needed to address these issues and, importantly, to put those results into the context of miRNA regulation.

### In silico analysis of miRNA: mRNA interactions in the adult mouse brain

**Figure 4:**
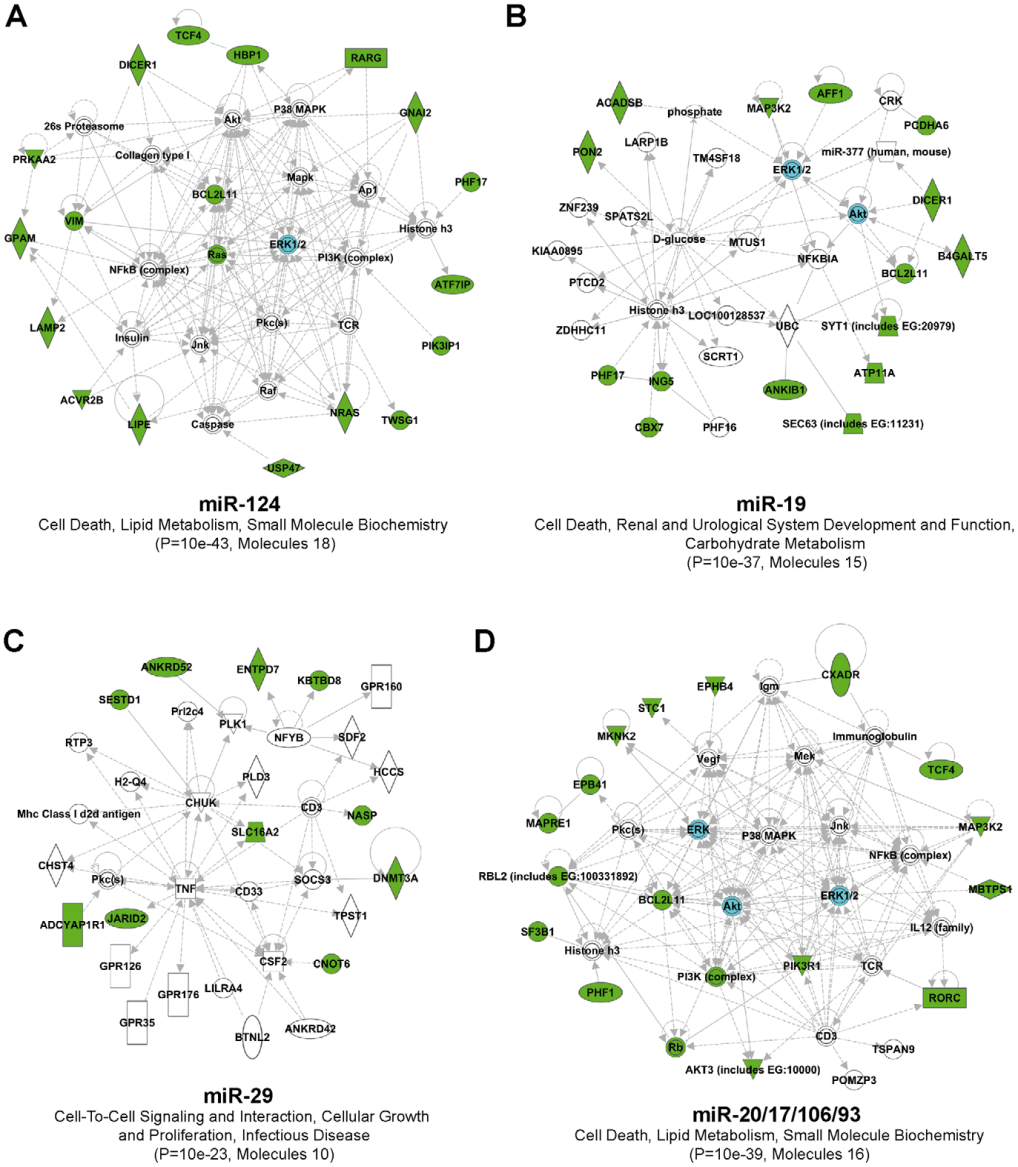
IPA networks associated with high-ranking seed sequences. The top-ranking biological networks associated with (**A**) miR-124, (**B**) miR-19, (**C**) miR-29 and **(D**) miR-20/17/106/93 predicted target genes are depicted. Related biological functions and P values are indicated and generated using the IPA software. Genes in green are significantly misregulated according to our microarrays. Genes in blue have been validated at the protein level in our previous study [14]. Images and P values were generated using the IPA software. Please refer to Figure S1 for further details.

Given the fact that miRNAs can promote mRNA degradation, we hypothesized that a subset of upregulated genes would comprise *bona fide* miRNA targets. In attempt to identify those targets, as well as effector miRNAs, we used the Partek Genomics Suite v6.6beta software (see materials and methods) to search for overrepresented miRNA seed sequences (hereafter refereed as seeds). Using this strategy, we identified 113 distinct seeds, originating from 227 miRNAs, which were significantly (P<0.05) enriched in the 3′UTR of upregulated genes (Table S5). We identified at least one miRNA target site in 62% (185 out of 299) of upregulated genes, with an average of 3.4 miRNA target sites per gene (data not shown). The ten most significant seeds, in combination with their predicted target genes, are listed in Table S6. The highest-ranking IPA networks associated with miR-124, miR-19, miR-29 and miR-20/17/106/93 predicted targets are depicted in Figure 4 and Figure S1. Abnormal regulation of these networks could have important biological consequences in the neuron. Notably, only 11 seeds, originating from 12 miRNAs, reached significance in our set of downregulated genes (Table S5). Thus, and consistent with our hypothesis, loss of Dicer activity *in vivo* is associated with an overall increase in putative miRNA target genes. It should be cautioned, however, that while several brain miRNAs (e.g., miR-124, miR-29, miR-134, miR-107, miR-9) are, as expected, downregulated in the neuronal *Dicer* cKO mice [33], not all miRNAs are quantitatively reduced in this model, as denoted recently by Babiarz *et al*. using RNA deep sequencing [34].

**Figure 5:**
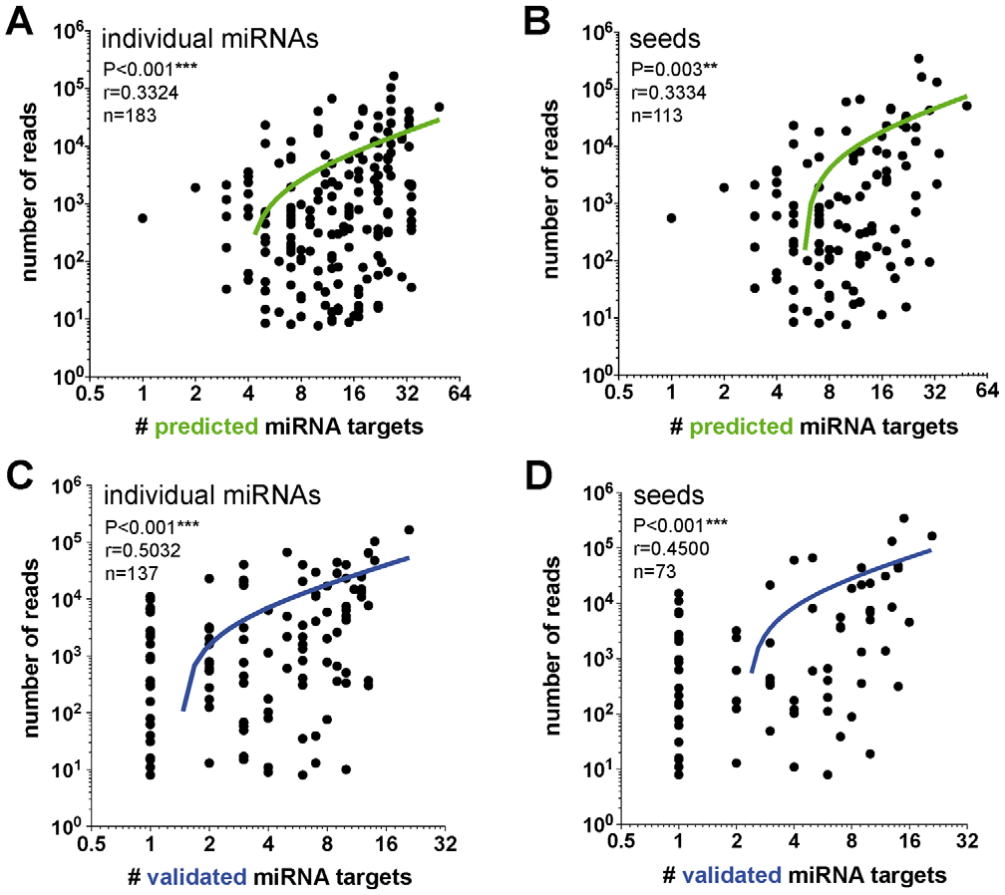
miRNA activity is associated with miRNA abundance. **(A, B**) Correlation between miRNA quantity and number of seeds with the number of predicted miRNA targets. *y*-Axis  =  log2(x), *x*-axis  =  log10(x). **(C, D**) Correlation between miRNA quantity and number of seeds with the number of validated miRNA targets. For these calculations, we used miRNAs with at least one validated target gene. *y*-Axis  =  log2(x), *x*-axis  =  log10(x).

To further investigate the functional relevance of our findings, we compared our results with those obtained from Babiarz *et al*. [34], where the authors quantified 365 mature miRNA species in the brains of ∼5 week-old wildtype mice (Table S7). Eighty-one percent (183 out of 227) of the miRNAs identified in our bioinformatics analyses were expressed in the brain, further strengthening our *in silico* predictions. By extension, these results suggest that a large fraction of the neuronal miRNome participates in the regulation of gene expression *in vivo*. Spearman correlation analysis demonstrated a significant (P<0.001, r = 0.3324, n = 183) positive relationship between miRNA abundance (i.e., number of reads) and number of predicted mRNA targets (Figure 5A). A similar correlation was observed when using the numbers of seeds (P = 0.003, r = 0.3334, n = 113) (Figure 5B). We performed a similar analysis using validated miRNA targets. To this end, validated miRNA targets (mouse) were extracted from the miRWalk database (http://www.umm.uni-heidelberg.de/apps/zmf/mirwalk). As before, we observed similar correlations between the numbers of reads of individual miRNAs (P<0.001, r = 0.5032, n = 137) or seeds (P<0.001, r = 0.4500, n = 74) and validated miRNA targets (Figure 5C, D). It should be noticed, however, that only a few validated miRNA targets were present in our list of misregulated genes (Table S7 and data not shown). Taken together, these results suggest that miRNA “activity” (i.e., mRNA targeting capability) is directly associated with miRNA quantity (i.e., abundance).

**Figure 6:**
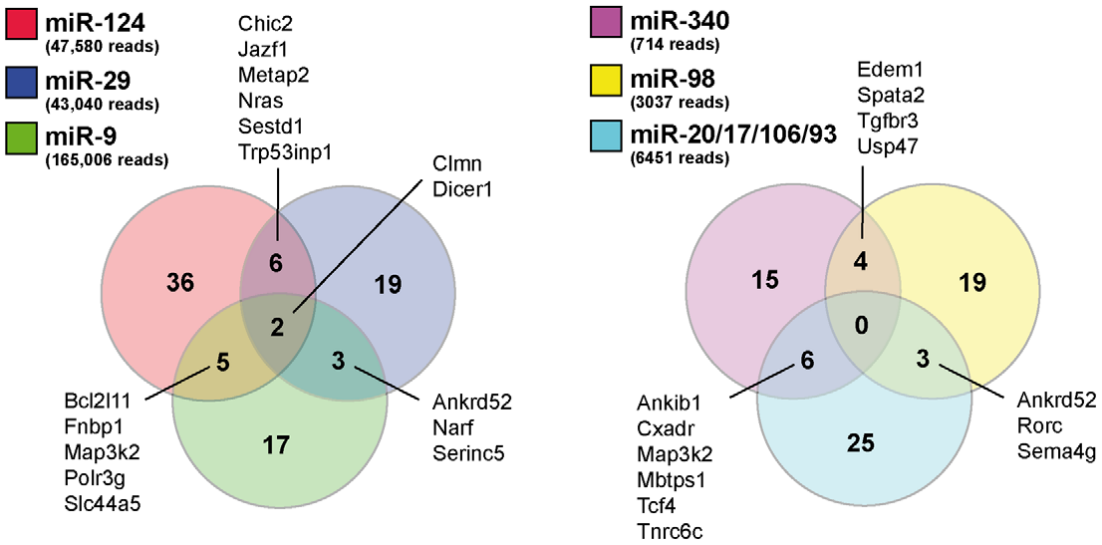
Overlapping miRNA targets. Diagram showing that highly (between 43000 and 165000 reads, left panel) and moderately (between 700 and 6500 reads, right panel) expressed miRNAs have overlapping mRNA targets. Overlapping predicted target genes are annotated.

We also confirmed the fact that some miRNAs had overlapping mRNA targets. This is exemplified using three highly (reads) and moderately (reads) expressed miRNAs (Figure 6). For this analysis, we focused on predicted miRNA targets. We did not observe, however, an enrichment in miRNA target site number within these or other mRNA transcripts (data not shown), suggesting that miRNAs with single target sites work in combination with other miRNAs to regulate the target mRNAs. Overall, these results strengthen the notion of “cooperativity” and “multiplicity” modes of miRNA regulation [7], [35], [36].

## Discussion

In this study, we analyzed in detail transcriptional profiles of mice lacking functional *Dicer* and miRNAs in post-mitotic forebrain neurons. These mice provide a unique and unbiased model to study miRNA-dependent gene networks and pathways under physiological conditions *in vivo*. On one hand, our results demonstrate that gene networks associated with various neuronal functions are downregulated in the absence of Dicer. On the other hand, upregulated genes were associated with biological processes such as gene expression regulation. We found that more than half of upregulated transcripts likely represent novel *bona fide* miRNA targets. Several key genes involved in the miRNA machinery, including the RISC core component eif2C2/Ago2, were increased in *Dicer* compromised brain, likely resulting from a loss of functional feedback loop or an attempt from the neuron to overcome the lack of miRNA abundance. Finally, several pathways associated with neurological diseases, neurological development, as well as cellular function and organization, were altered in the *Dicer* cKO mice. Taken together, our results strengthen the role for known miRNAs, such as miR-124, in neuronal identity and maintenance, but also suggest that most of the neuronal miRNome is important for neuronal homeostasis in mice and perhaps other mammals, including humans.

In attempt to better understand the molecular networks dependent on miRNA activity in the adult brain, we performed microarray data mining as well as various *in silico* analyses of misregulated genes in the *Dicer* cKO mice. These genome-wide analyses are necessary to fully grasp the molecular complexities associated with miRNA-dependent gene expression regulation. Our results point to a number of biologically important cellular functions that depend on miRNA activity in differentiated neurons in mammals. Some of these pathways are directly related to the neuronal shrinkage phenotype previously associated with cortical *Dicer* loss. Multiple other networks associated with autophagy, neurotransmission and neuronal death were also affected in this model. On this line of thought, several terms associated with neurological disorders were significant in the *Dicer* mutant mice, including *Huntington's disease* (P = 2.04e–9), *Alzheimer's disease* (P = 4.54e–3) and *Amyotrophic lateral sclerosis* (P = 1.47e–4), all ranking in the top fifteen of genetic disorders. Taken together, the results provide a good platform for further validation studies aimed at understanding specific miRNA-dependent pathways *in vivo*, both in health and in diseases.

While a significant enrichment in miR-124 and various other miRNA seeds were observed in the absence of neuronal *Dicer*, we observed no particular enrichment in validated miRNA target genes in our subsets of misregulated genes (Table S7 and data not shown). Several factors may explain these discrepancies, including data analysis biases, the stage of neuronal differentiation, cell type specificity, gain *vs.* loss-of-function paradigms, compensation mechanisms, or the unique *in vivo* neuronal milieu [37], [38]. While further studies are needed to clarify these possibilities, our study supports the hypothesis that neuronal *Dicer* and the miRNA pathway function to repress a subset of nonneuronal genes and to control a unique set of neuronal genes *in vivo.* It's noteworthy, however, that certain validated targets were indeed identified herein (e.g., Dnmt3a, Limk1), and could contribute significantly to neuronal integrity and maintenance *in vivo*. The challenge now is to understand the nature of such differences and possibly reassess the notion of “validated” miRNA target, at least in a certain physiological or living organism context. Novel techniques, such as the RIP-Chip assay [39], [40], will likely help to elucidate the number and identity of physiological miRNA target genes in the brain. It is also noteworthy that our microarray analyses do not take into account potential post-transcriptional changes in miRNA target genes. Acetylation of histones, for example, can change the conformation of the chromatin and consequently the transcription of certain genes. This could explain, at least in part, the rather low overlap of validated miRNA targets in our datasets. Thus, combined transcriptome and proteomic analyses will be required to fully assess the number and identity of genes regulated by neuronal miRNAs *in vivo*.

Combined with previous literature, our studies suggest that miR-124 likely functions as a “molecular hub” in neuronal specification, function and maintenance. However, our *in silico* and bioinformatics analyses strongly suggest that additional miRNAs, up to 50% of the neuronal miRNome, function in concert with miR-124 to fine-tune neuronal functions and homeostasis. One example is the proposed role for miR-132 (seed sequence P = 8.18e–4) in synapse formation and arborization [41]–[43]. Another example includes miR-29 (seed sequence P = 5.04e–08) involved in neuronal apoptosis [44].

It is noteworthy that predicted miR-124 target genes are expressed at rather low levels (1.43x ±0.22) in the *Dicer* cKO mice (data not shown). This observation is in agreement with previous studies in cultured cells [20], [45], suggesting that abundant miRNAs are involved in the widespread regulation of weakly expressed genes. From the observations herein, this rule seems also to apply for less abundant miRNAs. This raises the question of the relationship between miRNA and mRNA abundance and activity. While our bioinformatics analysis strengthens the multiplicity and the cooperative modes of miRNA action, the physiological reason for this level of complexity remains unclear. The more common “one miRNA-one target” concept is thus extremely over-simplistic. Obviously, miRNA gene knockout models are required to fully assess the physiological relevance of our experimental system. In addition, a mechanism of compensation cannot be excluded at this stage of investigation.

Interestingly, REST expression and downstream effectors were not significantly affected by the ablation of Dicer activity in the adult brain (Figure S3), suggesting that this transcription factor does not play a major role in neuronal maintenance, at least at the age and in the mouse model presented herein. We would like to point out that negative data are not conclusive, and REST may have more limited, precise functions in the adult brain, as suggested by the changes in a small number of REST-dependent genes (Figure S3). Moreover, REST functions go beyond the transcriptional regulation of neuronal genes. Indeed, REST is also involved in mediating dynamic interactions between genomic organization, nuclear architecture, and transcription in a developmentally regulated and environmentally responsive manner [46]. REST also associates with various co-regulators, including histone deacetylases and with DNA methyltransferases, one of which is misregulated in our *Dicer* cKO model (Dnmt3a).

In conclusion, our study provides a first comprehensive view of the effects of *Dicer* ablation in mammalian post-mitotic neurons in vivo. These effects are likely different from differentiating neurons *in vivo* and differentiated neurons *in vitro*. While *Dicer* deficiency is a somewhat crude experimental approach, these mice provide a unique model to explore the possible collaborative effects of miRNAs on mRNA targeting in a physiological context. Clearly, future experiments are required to understand the relationship between miRNAs and their target genes in the brain.

## Materials and Methods

### Animals

The generation and characterization of the forebrain-specific *Dicer* cKO mice (CamKII-*Cre*/+; *Dicer* ^flox^/^flox^) and control (CamKII-*Cre*/+; *Dicer* ^flox^/^+^) mice was described previously [14]. Unless otherwise stated, 9–10.5 week-old *Dicer* cKO and littermate controls were used (3 from each group). The CRCHUQ-CHUL ethical committee approved all animal studies.

### RNA extraction, quantitative RT-PCR and standard PCR

Total RNA was extracted from cells and brain using the miRVana PARIS kit (Ambion) according to manufacturer's instructions. Real-time quantitative PCR was carried out as described [47], [48]. Primer sequences are listed in the Table S2. Relative expression was calculated by using the comparative CT method. Gapdh was used as normalization control, as described [48].

### Protein analysis

Immunohistochemistry was carried our as before [14] using Nefl (Abcam, #ab7255) antibodies. Western blot analysis was performed as before [14] using Nefl, Nefh/SMI31 (Covance, #SMI-31R), and Gapdh (Millipore, #MAB374) antibodies.

### Microarray and data analysis

Microarray analyses were carried out as before [48] using Mouse Exon 1.0 ST arrays (Affymetrix). Briefly, total RNA (200 ng per sample) was labeled using the Affymetrix GeneChip® WT cDNA Synthesis and Amplification Kit protocol and hybridized to the arrays as described by the manufacturer (Affymetrix, Santa Clara, CA). The cRNA hybridization cocktail was incubated overnight at 45°C while rotating in a hybridization oven. After 16 hours of hybridization, the cocktail was removed and the arrays were washed and stained in an Affymetrix GeneChip fluidics station 450, according to the Affymetrix-recommended protocol. The arrays were scanned using the Affymetrix GCS 3000 7G and the Gene-Chip Operating Software (Affymetrix, Santa Clara, CA), to produce the intensity files. The background subtraction and normalization of probe set intensities was performed using the method of Robust Multi-array Analysis (RMA) described by Irizarry *et al.* [49]. To identify differentially expressed genes, gene expression intensity was compared using a moderated *t*-test and a Bayes smoothing approach developed for a low number of replicates [50]. To correct for the effect of multiple testing, the false discovery rate, was estimated from *p* values derived from the moderated *t*-test statistics [51]. The analysis was performed using the Partek Genomics Suite software (http://www.partek.com/partekgs). Overrepresented miRNA target sites were identified using the “MicroRNA Integration” application and the TargetScanMouse 5.2 database (http://www.targetscan.org/mmu_50). Further details are available on demand.

### Pathway and network analysis

The list of significant Dicer-dependent genes identified by Partek Genomics Suite, containing Affymetrix probe set IDs, fold changes and p values, were uploaded into the Ingenuity Pathway Analysis (IPA) tool (www.ingenuity.com). Each clone identifier was mapped to its corresponding gene object in the Ingenuity Pathway Knowledge Base (IPKB). These so-called focus genes were then used for generating biological networks, using the “IPA Core Analysis” function. To start building networks, the application queries the IPKB for interactions between focus genes and all other gene objects stored in the knowledge base, and generates a set of networks. Every resulting gene interaction has supporting literature findings available online. IPA then computes a score for each network according to the fit of the user's set of significant genes. The score is derived from p-value and indicates the likelihood of the focus genes in a network being found together as a result of random chance. A score of 2 indicates that there is a 1-in-100 chance that the focus genes are together in a network as a result of random chance. Therefore, scores of 2 or higher have at least a 99% confidence of not being generated by random chance alone.

### GO term analysis

GO term analysis of misregulated genes (P<0.05, FDR <0.25) was performed using the *Database for Annotation, Visualization and Integrated Discovery* (DAVID) version 6.7 (http://david.abcc.ncifcrf.gov/).

### Statistical tests

Unless otherwise indicated, all statistical tests were performed using the GraphPad Prism® version 5.0b software.

## Supporting Information

Figure S1 **Legend for gene networks and canonical pathways generated by the Ingenuity Pathway Analysis** (**IPA**) **designer tool.** (**A**) Network shapes are shown. They include proteins such as cytokines, growth factors, enzymes and different regulators and receptors. (**B**) Relationship types are described. Solid and dotted lines imply direct and indirect relationships between proteins, respectively. “Acts on” and “inhibits” edges may also include a binding event.
(TIFF)

Figure S2 **No changes in Nefh.** Immunohistochemistry of Nefh in the cortex of control and *Dicer* cKO mice. No significant changes in Nefh signal (in green) were observed (highlighted in white square). In this example, we used a 13 week-old *Dicer* cKO mice and age-matched control. Overall, these results are consistent with the microarrays.
(TIF)

Figure S3 **REST expression levels in the absence of Dicer in the adult brain.** (**A**) Real-time qRT-PCR shows no significant expression changes of murine REST mRNA in the cortex of *Dicer* cKO mice (n = 3 per group, p = 0,1403, *Student unpaired t* test). (**B**) REST protein expression levels remain stable in the absence of neuronal Dicer in vivo (n = 3 per group). Samples were taken from previous studies [33], [47]. β-Actin was used as loading control. (**C**) Endogenous REST protein levels decrease in mouse Neuro2A cells transfected 48 hours with pre-miR-124 (50 nM), demonstrating the specificity of our REST antibody. Samples were taken from a previous study [47]. β-Actin was used as loading control. (**D**) No significant enrichment of the misregulated genes (n = 755) in the REST network (n = 153) as determined by IPA (9 molecules, p = 0,1337). Molecules in green and in red are upregulated or downregulated, respectively, as determined by our microarrays.
(TIF)

Table S1 **List of significantly affected genes in the cortex of adult Dicer cKO compared to control mice.**
(XLSX)

Table S2 **Primers used for real-time qRT-PCR.**
(XLSX)

Table S3 **Functional analysis of misregulated genes in** ***Dicer*** **cKO compared to control mice.**
(XLSX)

Table S4 **Molecular pathways associated with neuronal miRNA loss in** ***Dicer*** **mutant mice.**
(XLSX)

Table S5 **Significantly enriched miRNA seed sequences in the 3′UTRs of misregulated genes.**
(XLSX)

Table S6 **Top ten of significant miRNA seed sequences and their respective target genes.**
(XLSX)

Table S7 **Comparison of the overrepresented seed sequences with published deep sequencing data.**
(XLSX)
