# Supplementary figures and images for: Gene Network and Pathway Analysis of Mice with Conditional Ablation of Dicer in Post-Mitotic Neurons
Source: PLoS One. 2012 Aug 27;7(8):e44060. doi: 10.1371/journal.pone.0044060 (PMC3428293; doi:10.1371/journal.pone.0044060)

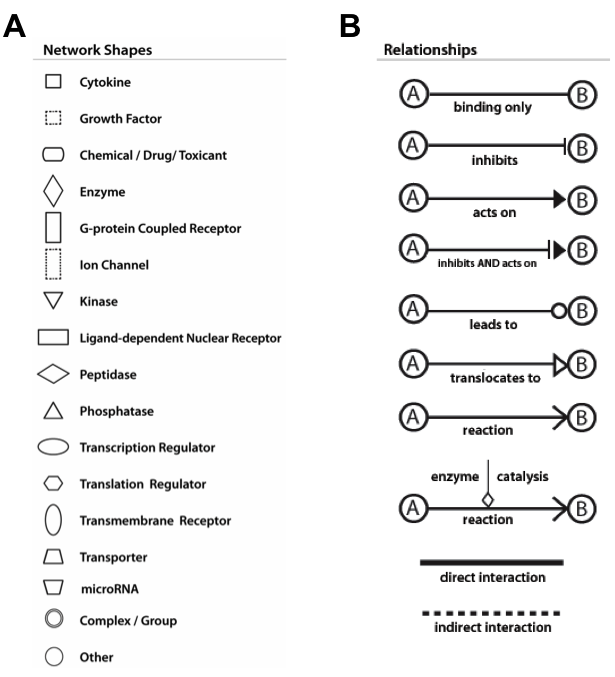

Supplement: Figure S1 — Legend for gene networks and canonical pathways generated by the Ingenuity Pathway Analysis (IPA) designer tool. (A) Network shapes are shown. They include proteins such as cytokines, growth factors, enzymes and different regulators and receptors. (B) Relationship types are described. Solid and dotted lines imply direct and indirect relationships between proteins, respectively. “Acts on” and “inhibits” edges may also include a binding event. (TIFF) [file pone.0044060.s001.tiff]

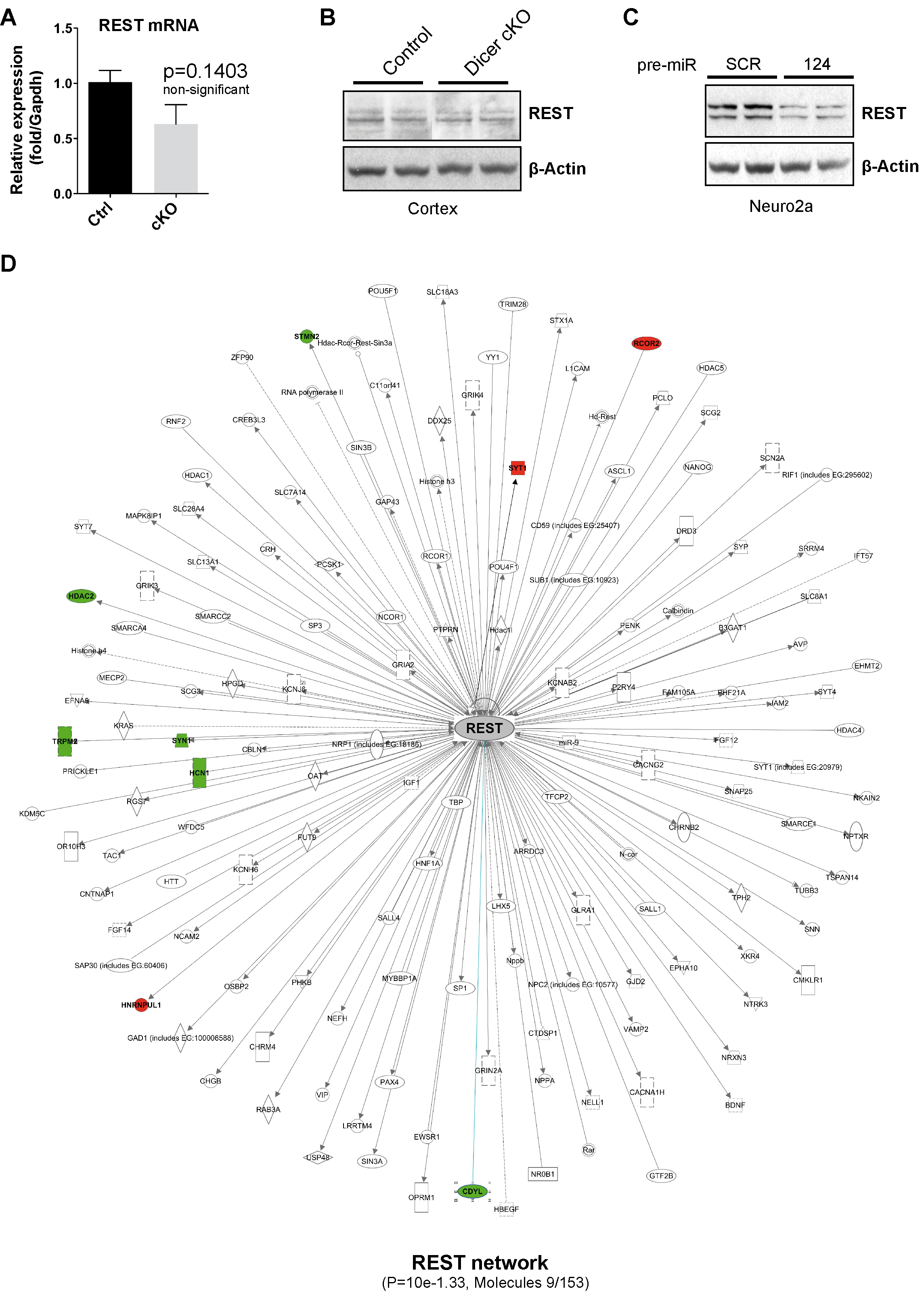

Supplement: Figure S2 — No changes in Nefh. Immunohistochemistry of Nefh in the cortex of control and Dicer cKO mice. No significant changes in Nefh signal (in green) were observed (highlighted in white square). In this example, we used a 13 week-old Dicer cKO mice and age-matched control. Overall, these results are consistent with the microarrays. (TIF) [file pone.0044060.s002.tif]

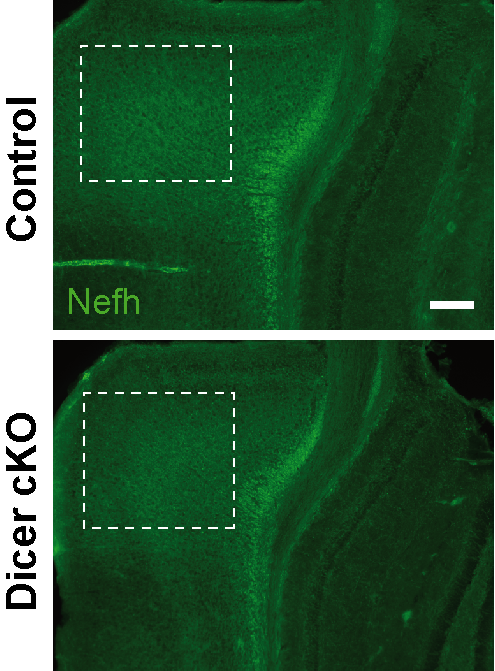

Supplement: Figure S3 — REST expression levels in the absence of Dicer in the adult brain. (A) Real-time qRT-PCR shows no significant expression changes of murine REST mRNA in the cortex of Dicer cKO mice (n = 3 per group, p = 0,1403, Student unpaired t test). (B) REST protein expression levels remain stable in the absence of neuronal Dicer in vivo (n = 3 per group). Samples were taken from previous studies [33], [47]. β-Actin was used as loading control. (C) Endogenous REST protein levels decrease in mouse Neuro2A cells transfected 48 hours with pre-miR-124 (50 nM), demonstrating the specificity of our REST antibody. Samples were taken from a previous study [47]. β-Actin was used as loading control. (D) No significant enrichment of the misregulated genes (n = 755) in the REST network (n = 153) as determined by IPA (9 molecules, p = 0,1337). Molecules in green and in red are upregulated or downregulated, respectively, as determined by our microarrays. (TIF) [file pone.0044060.s003.tif]
